# Medical Notes of Travel

**Published:** 1855-01

**Authors:** 


### MEDICAL NOTES OF TRAVEL.

Our recent visit to the eastern cities, in the main very agreeable and pleasant, was also more or less profitable. It furnished us with an opportunity of observing the medical typography of different localities, although intermediate points were traversed with the rapidity of flight itself, rendering observation both impracticable and impossible.

The long protracted and great intensity of the heat of the past summer, attended with a withering drought, left its effects upon many portions of the country through which we passed. In these localities dysentaries, fevers, and gastro enteric diseases of children, were the ordinary inflictions. In several places the cholera had prevailed, and in others it continued to exist. Pittsburgh suffered severely by that disease, more so than ever before. The citizens were lulled into security because it was supposed that there were ever present with them, agents competent to protect them.—But their sulphurous emanations thrown into the air from their hundreds of chimnies of forges, rolling-mills, foundries, glass-works, and manufactories in which bituminous coal is burned even in midsummer, failed this last season in neutralizing the epidemic influence there, and throughout the extent of the country, or of destroying the pestiferous emanations welling up from their foul and stagnant line of canal, containing vegetable and animal matters in active decomposition. These emanations, when added to this prevailing influence, quickened it into activity and rendered it more potent for deadly mischief. Although these numerous stacks of chimnies belched forth night and day their lurid flames and filled the air with columns and clouds of smoke, and athough the city authorities ignited masses of coals upon the surface of the ground with the view to the purification of the atmosphere, the disease continued to progress until many of their most prominent and useful citizens were borne away to their final resting place. It continued to linger there even to the first of the present month, as a few cases were then in the city or county prison.

The course pursued by the board of health was truly singular and extraordinary for men of intelligence. We were informed that they were in the habit, once in twenty-four hours, of opening pipes communicating with the reservoir, and permitting water to flow into this pest slough just sufficient to disturb the filthy deposits and keep it liquid, and therefore more active and potent for mischief. Foul as it had become in consequence of the want of water, and however unfortunate its presence may have proved, yet a little reflection would have taught them the true and humane policy of keeping it in a state of rest, and allowing it if possible to evaporate to dryness. With an almost impenetrable cloud of smoke above, a plague driven through its midst, a deadly pestilence destoying the inhabitants by hundreds, the sooty blackness of the buildings, looking for all the world like a funeral pall spread over it, the rivers drained of their waters, their city must have presented a picture painfully drear and gloomy.

Columbia, lying west of Philadelphia, suffered more than any other place in proportion to the number of inhabitants, during the season from the same disease. The cause was found to be in a large pool of stagnant water, into which a number of dead animals had been thrown. Here, as at Pittsburgh, the disease entered the abodes of the wealthy and others in comfortable circumstances, and made there numerous victims. Dismay and alarm seized upon the minds of the people, and numbers sought safety in flight.—Such was the fearful character of its visitation, that the citizens fled in such numbers as to leave the town almost deserted. There is little doubt but that the panic lent potency to the causes already existing in the production of the disease, and increased the mortality. Elsewhere, in the different localities in which it appeared, it selected for its victims those generally whose habits of life rendered them fit subjects of disease of any kind. But at Pittsburgh and Columbia, it took its victims as well from the ranks of those who enjoyed the comforts of life, the temperate and the prudent, as the intemperate and filthy.

The profession mourns the loss of several of its members who fell while fighting manfully with the foe at Columbia. Faithful to their vocation, and true to humanity, they died at their posts of duty.

At Baltimore, we were informed by one of the distinguished Professors of the Medical University, Prof. Chew, that there was at the time a case of true Yellow Fever in that city. The Professor did not see the case himself, but had it from a most reliable source.

At Martinsburgh, Virginia, on the line of the Baltimore and

Ohio R. R., and east of Cumberland, the Cholera was there also. This arose from the stagnant water in the C. & O. Canal, holding in suspension vegetable and animal matters.

At Cumberland we took the coach and crossed the mountains to Uniontown, Pa. Along the route our attention was frequently called to some mountain specimen of humanity, whose sallow icterode look indicated an intermittent. This occurred so frequently that we were led to enquire into the cause, when our supposition was fully confirmed. We were somewhat astonished to find these cases so numerous in these high mountain regions. We had traversed these very often in times past, but we never saw so many cases of purely intermittent there before. The physical aspects of the people generally through these regions were unfavorable, and would not compare in vigor, freshness, and other evidences of health with the people of the west. They impressed us with the idea that they were a decaying and declining race, and far, very far, from filling the ideal picture we have been in the habit of drawing of the "hardy mountaineers." A gentleman was pointed out to us as a physician who was plodding along through these giddy heights, and it occurred to us that for his extraordinary labors, his exposures, his devotion, and his more than monkish seclusion, away from social refinement, he should have a pension settled upon him for life. We descended from these rugged mountain heights into the rich and fertile valley below, in which is situate, and which is to be seen from a point in the mountain, the village of Uniontown.

During our stay here, we learned that the place had been very unhealthy during the hot summer months. A medical gentleman there, Dr. Patrick, informed us that as many as forty children had died from gastro-enteritis, in a population of a little over three thousand inhabitants; a mortality which would frighten the citizens of our western cities and towns.

There were cases of fever, which from the description, were of a low type of continued fever; perhaps the ataxo-adynamic, as described by Andral.

While wandering through the streets of this place, now more than a half a century old, the great number of old and decaying wooden houses attracted our attention. An enquiry suggested itself touching the influence these decaying tenements might exert over the health not only of the occupants, but the citizens at large. The gaseous exhalations from the decomposition of ligneous or woody matters are mainly carboniferous, well known to be deleterious to health, and in contracted forms, destructive to life; and may not certain endemical visitations in certain localities of cities and towns often depend upon this as the cause. The form of fever termed ship-fever, is believed to proceed from the poisonous emanations arising from the dry decomposition of the hulls of ships. We have seen instances ourselves, which would go far to sustain this position. If so in this instance, why not affect the health of the inmates as well as of those living in the midst or in the neighborhood where a majority of the houses and other structures were under the dominion of a rapid decay and decline?

This subject is worthy the consideration of the "*fathers*" of those old towns whose houses are now decayed, and if annual visitations of some form of disease takes place exclusively in those localities, then they should be abandoned or the cause removed.

